# The Use of Quantile Regression to Forecast Higher Than Expected Respiratory Deaths in a Daily Time Series: A Study of New York City Data 1987-2000

**DOI:** 10.1371/journal.pone.0078215

**Published:** 2013-10-11

**Authors:** Ireneous N. Soyiri, Daniel D. Reidpath

**Affiliations:** 1 South East Asia Community Observatory (SEACO), School of Medicine and Health Sciences, Monash University, Kuala Lumpur, Malaysia; 2 Global Public Health, School of Medicine and Health Sciences, Monash University, Kuala Lumpur, Malaysia; National Institutes of Health, United States of America

## Abstract

Forecasting higher than expected numbers of health events provides potentially valuable insights in its own right, and may contribute to health services management and syndromic surveillance. This study investigates the use of quantile regression to predict higher than expected respiratory deaths.

Data taken from 70,830 deaths occurring in New York were used. Temporal, weather and air quality measures were fitted using quantile regression at the 90th-percentile with half the data (in-sample). Four QR models were fitted: an unconditional model predicting the 90th-percentile of deaths (Model 1), a seasonal / temporal (Model 2), a seasonal, temporal plus lags of weather and air quality (Model 3), and a seasonal, temporal model with 7-day moving averages of weather and air quality. Models were cross-validated with the out of sample data. Performance was measured as proportionate reduction in weighted sum of absolute deviations by a conditional, over unconditional models; i.e., the coefficient of determination (R1).

The coefficient of determination showed an improvement over the unconditional model between 0.16 and 0.19. The greatest improvement in predictive and forecasting accuracy of daily mortality was associated with the inclusion of seasonal and temporal predictors (Model 2). No gains were made in the predictive models with the addition of weather and air quality predictors (Models 3 and 4). However, forecasting models that included weather and air quality predictors performed slightly better than the seasonal and temporal model alone (i.e., Model 3 > Model 4 > Model 2)

This study provided a new approach to predict higher than expected numbers of respiratory related-deaths. The approach, while promising, has limitations and should be treated at this stage as a proof of concept.

## Introduction

There is an increasing body of literature looking at the causal relationship between weather, air quality factors, and health outcomes [1-4]. Forecasting health outcomes has attracted less attention, but it too has a developing base in the scientific literature [5-9]. Traditionally, both the causal modelling and the forecast research have focused on the central tendencies of the distribution of data; i.e., the expected and conditional expected value. For instance, a typical generalised linear model of daily COPD events will model the expected number of COPD cases each day conditioned on a series of weather, air quality, and perhaps individual factors [10,11].

Although the expected outcomes can be important, the central portion of the conditional distribution is only one part of the story, and other parts of the conditional distribution can give quite different insights – particularly when the distributions are skewed. Studies of birth weight for example have shown quite different relationships between the explanatory variables and birth weight when modelling the conditional mean than they have when modelling low birth weight, such as birth weights in the lowest decile [12]. There is no requirement for the factors explaining low birth weight to be the same factors that explain average birth weight or for the explanation to be of the same form as for the central part of the conditional distribution.

Similarly, in modelling daily respiratory events (morbidity or mortality) and their relationship to air quality or weather, there is no strong requirement for the relationships that model average events to be the same as the relationships that model days with unusually high or unusually low numbers of events. By extension, forecasting the numbers of respiratory events on the outer arms of a conditional distribution need not rely on the same predictors that would be useful in forecasting the expected number of respiratory events.

To our knowledge, and not withstanding its potential value, there has only been one study looking at the forecasting of the number of respiratory events on the outer arm of a conditional distribution (such as the 90^th^ percentile)[13]. There is the simple scientific interest in our capacity to make such forecasts, and what insights it might provide into the data; but there is also potential value for forecasting likely resource needs, as well as in areas such as syndromic surveillance, where the number of events exceeding a threshold is used to trigger a health systems response. Quantile regression remains a relatively unusual modelling technique in health research, which can be used to model conditional responses at any quantile of interest; and – although it has been used (rarely) for forecasting [14,15] – to our knowledge has never been used to forecast mortality.

## Methods

We investigated the use of quantile regression to forecast the 90^th^ percentile of daily, respiratory related deaths for New York City, in the period 1 January 1987 to 31 December 2000. The choice of the 90^th^ percentile was somewhat arbitrary but in keeping with the idea of understanding the general capacity that a health system might need to maintain to meet typical demand. The data were drawn from the National Morbidity, Mortality, and Air Pollution Study (NMMAPS)[16], which are publicly available data through the Health and Air Pollution Surveillance System website (http://www.ihapss.jhsph.edu), and, in our case, accessed using the NMMAPS package in the R statistical environment [17]. The daily count of respiratory deaths was the outcome measure of interest. The data included 70,830 respiratory deaths over 5,114 days of surveillance. 

The dataset also included a range of daily weather and air quality measures which were used as predictors in the modelling. The predictors included daily mean air temperature, dew point, ozone (O_3_), sulphur dioxide (SO_2_), nitrogen dioxide (NO_2_), and carbon monoxide (CO). Measures of particulate matter were not included because of the levels of missing data within the dataset. In addition to the measures of weather and air-quality, cosinor values representing a yearly and a half yearly cycle[18,19], and dummy variables representing the days of the week were also used as predictors.

The data were sub-divided into two equal sized sets, from 1 January 1987 to 31 December 1993 for model development (*in-sample*), and from 1 January 1994 to 31 December 2000 for cross-validation (*out-sample*). We used the terms “prediction” to refer to the *in-sample* model development and then “forecasting” to refer to *out-sample* cross-validation. The size of the *in-sample* data was subsequently reduced to 2405 days (94.0% of total days) because of the use of lagged data, and a small amount of pre-existing missing data. The *out-sample* data (2548 days) were almost complete with a loss of only 8 days of data.

The details of quantile regression have been described elsewhere[20,21], as has its application to health problems[22,23]. The use of quantile regression with count data is unusual and its application to health forecasting remains novel [24,25].

A common challenge in modelling outcomes related to environmental exposures is the lagged effect between weather and air quality exposures and the health outcome of interest [26]. To identify an appropriate lag to represent the exposure to each of the weather and air quality measures, a series of quantile regression count models, using the *in-sample* data, were constructed testing the fit for each lag in turn, from a 1-day lag through to a 7-day lag; and was similar to approaches used elsewhere [9]. The fit of each lag, for each weather and air quality measure, was assessed using a function based on the asymmetric Laplace distribution commonly used in quantile regression. Best fit was determined by the lag that had the lowest value for:

$$\left[\left(\text{1}-p\right)\text{.}\sum_{y_{i}<q}\left|y_{i}-q\right|\right]+\left[p\text{.}\sum_{y_{i}>q}\left|y_{i}-q\right|\right]$$ (1)

where the absolute deviations below quantile *q* are weighted by 1- *p* if the actual values lies below *q*, and *p* if the actual value lies above *q*. The lags that were identified for inclusion were: CO, NO_2_, O_3_, dew point (1-day), temperature and SO_2_ (3-days). A 7-day moving average value for each of the weather and air quality factors was also included in one model as a point of contrast.

Four separate models were subsequently developed, three of which used quantile regression with either the selected lags or 7-day moving averages as predictors with the *in-sample* data. Model 1 was the intercept only model, an unconditional model predicting the value of the 90^th^ percentile of daily respiratory deaths to be constant across the data. Model 2 was a conditional model in which the value of 90^th^ percentile of daily respiratory deaths varied, conditioned on seasonal (cosinor values) and temporal (day of the week) predictors. Model 3 was a conditional model in which the value of 90^th^ percentile of daily respiratory deaths varied conditioned on seasonal/temporal predictors and the selected lags of weather and air quality predictors. Model 4 was the same as Model 3 except that the 7-day moving averages of weather and air quality predictors were used instead of selected lags.

The parameter estimates and standard errors from the quantile regression are not reported here, because they are essentially not required in the process for developing the forecasting model. Previous experience suggests that when they are provided, attention is inappropriately placed on that, rather than the predictive and forecasting capacity of the models.

The measure of fit used to establish the predictive validity (*in-sample* fit) and the forecasting accuracy (*out-sample* fit) of the quantile regression models was the coefficient of determination (R1)[20]. R1 measures the proportionate reduction in the weighted sum of the absolute deviations (WSADs) achieved by a conditional model over the unconditional model; where the weighted sum of the absolute deviations is given by equation 1. In this context, R1 is analogous to the mean absolute scaled error suggested by Hyndman and Koehler [27]. R1 was estimated for Models 2, 3, and 4, using the weighted sum of absolute deviations from Model 1 as the denominator. A supplementary approach which has been suggested for the application of count data in Quantile regression is for the data to be “jittered” prior to modelling [24]. In our preliminary work we did not observe any significant differences in model performance between the models of jittered data (of up to ±0.1 random numbers) and the time indicator, and those we reported.

## Results

**Figure 1:**
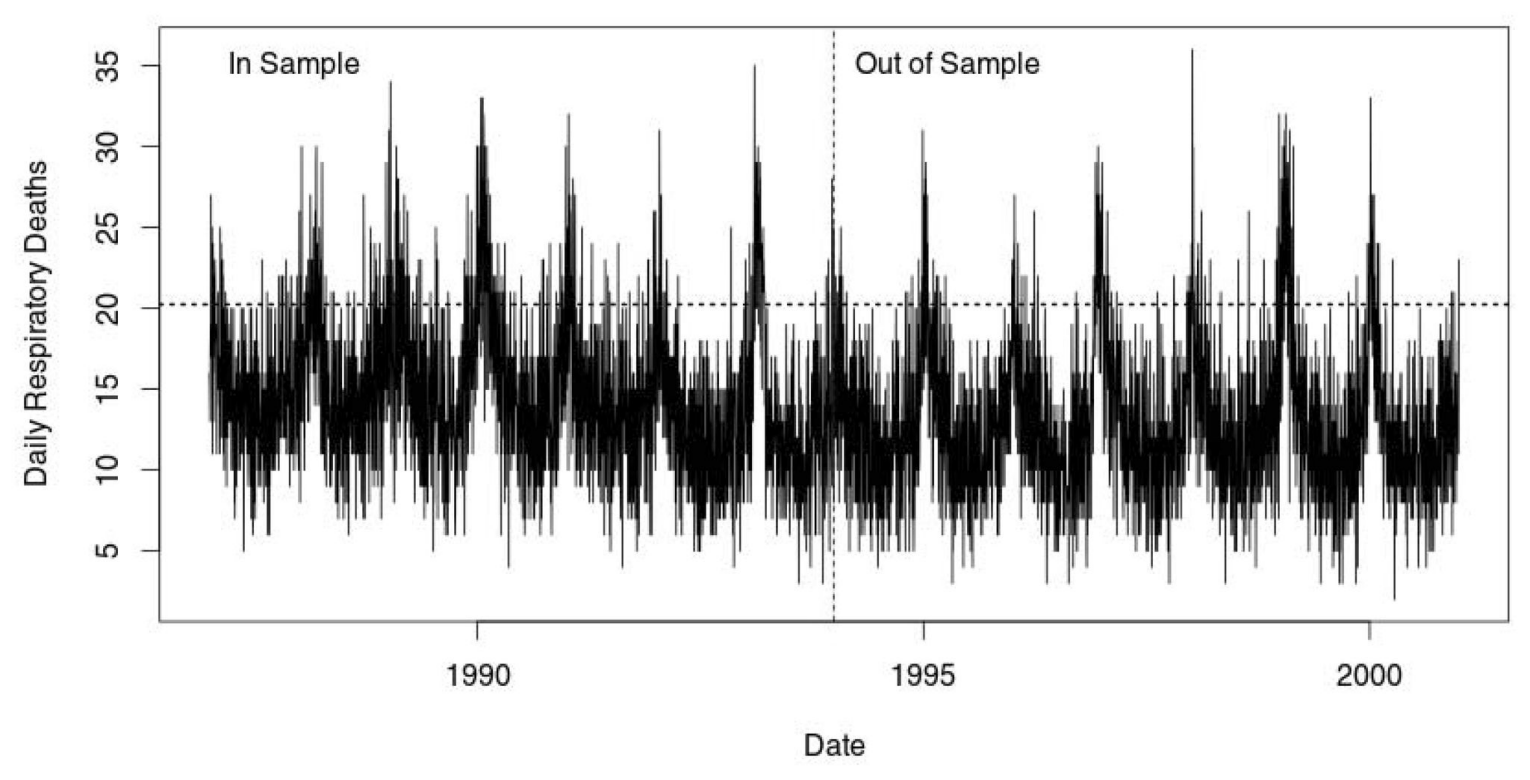
Time series of respiratory related deaths 1987—2000. The vertical dashed line indicates the separation between the in-sample and out-of sample data.

The time-series graph of the daily, respiratory deaths shows the familiar annual cycle with the peak deaths occurring in the winter months and the valleys occurring in the summer (Figure 1). The dashed vertical line in the middle of the figure shows the separation between the *in-sample* used to develop the quantile regression models and the *out-sample*, used to cross-validate the forecast models. The dotted horizontal line shows the *in-sample*, unconditional, 90^th^ percentile number of daily deaths (20.2 per day). It is clear that a conditional distribution at the 90^th^ percentile which included seasonal/temporal predictors would be quite different from the straight line of the unconditional quantile. 

The top half of Table 1 shows the predictive capacity of the three conditional models (Models 2, 3, and 4) relative to the unconditional model (Model 1). The coefficient of determination (R1) showed improvements between .165 (i.e., a 16.5% improvement in the fit) and .191 over the unconditional model. The seasonal model (Model 2) slightly outperformed both the selected lags model and the 7-day moving average model.

**Table 1:** A comparison of the predictive and forecasting capacity of the models.

	**Parameter**	**Model 1**	**Model 2**	**Model 3**	**Model 4**
**Prediction**					
In sample	WSAD	2328.2	1882.5	1943.3	1927.5
	R1	0	.191	.165	.172
**Forecasting**					
Out sample	WSAD	2529.5	2121.3	2039.7	2055.3
	R1	0	.161	.194	.187

Perdition was based on the In Sample (days=2445) and forecasting was based on cross-validation of the Out Sample (days=2548): Model 1, intercept only; Model 2, temporal/seasonal model, Model 3, temporal/seasonal model with selected lags of weather and air quality; and Model 4 temporal/seasonal model with a 7-day moving average of weather and air quality. The weighted sum of the absolute deviation (WSAD) and the coefficient of determination (R1) are used to compare the models.

The models developed using the *in-sample* data were cross-validated using the *out-sample* data. The lower half of Table 1 shows the forecasting capacity of the three conditional models (Models 2, 3, and 4) relative to the unconditional model (Model 1). As anticipated, the forecasting performance of Model 2 (seasonal/temporal predictors) was slightly worse than the predictive performance (R1=.161); i.e., cross-validation against the *out-sample* was slightly weaker than predictions based on the model development data (*in-sample*). Model 3 (seasonal/temporal predictors and selected lags for weather and air quality) performed slightly better in forecasting than it had in prediction (R1=.194). The performance of the 7-day moving average model (Model 4) also improved slightly (R1=.187).

**Figure 2:**
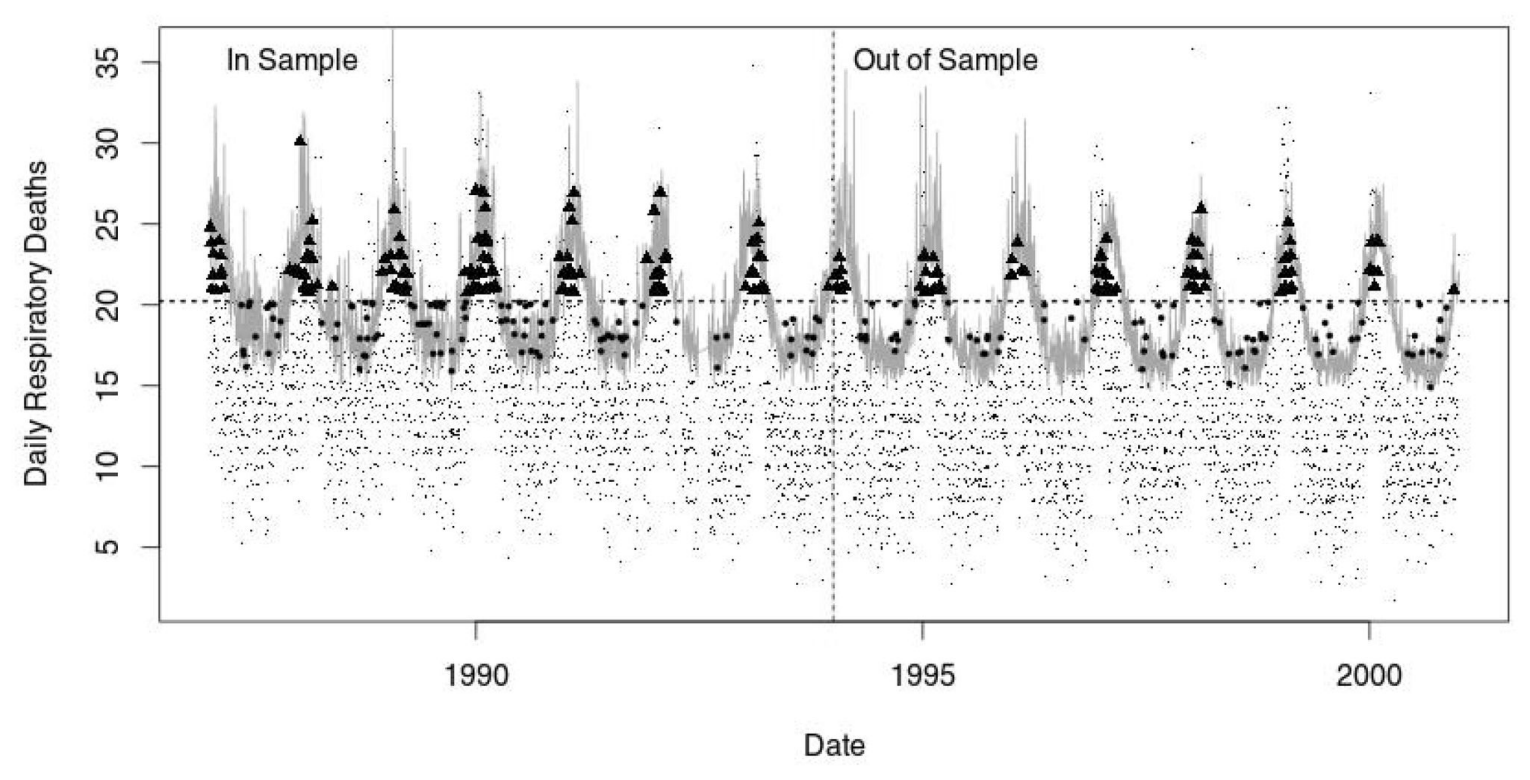
The quantile regression (90^th^ percentile) model of respiratory related deaths. The small dots indicate daily deaths. The dotted horizontal line shows the unconditional 90^th^ percentile.

As an illustration, Figure 2 shows the predictions (*in-sample*) and forecasts (*out-sample*) based on Model 3 (seasonal, temporal and selected lags as predictors) against the unconditional Model 1. Each point (small dot, larger dot, and solid triangle) represents the number of respiratory deaths recorded for a particular day. The conditional quantile regression model for the 90^th^ percentile (shown in grey) lies, as expected, well above the central portion of the data – where a model of the conditional mean or median would lie – and shows clear seasonal variation. The dotted horizontal line shows the unconditional 90^th^ percentile (Model 1). A few matters are worthy of note. All the points that lie above the dashed horizontal line would be regarded, under the unconditional model, as reflecting more unusual numbers of respiratory deaths. The points shown as black triangles reflect those days with numbers of deaths identified as more unusual under the unconditional model, but more typical under the more complex, conditional model. Conversely, all the points that lie below the dashed line would be regarded as more typical under the unconditional. The points shown as larger dots reflect those days with numbers of deaths identified as more typical under the unconditional model, but more unusual under the more complex, conditional model. Visually, the predictive and forecast performance of the model appears to be reasonably consistent (Figure 2).

## Discussion

The notion of modelling and forecasting the expected number of daily deaths is well described in the literature [2,28-30]. Forecasting any health outcome on the outer arms of a conditional distribution, however, is unusual [15], and appears not to have been done in the analysis of daily time series data related to mortality. This is unfortunate, because there are things to be learned from forecasts made at, for instance, the 90^th^ percentile that could not be learned from forecasting the expected number of daily deaths. 

For example, forecasts of the expected number of deaths will underestimate the kinds of resources that need to be available much of the time, particularly in an environment with the kind of variability shown in the daily respiratory deaths data. There is cyclical variation in the data, but even within the data at any one part of the annual cycle, there is substantial daily variation. Forecasts of likely numbers of deaths (i.e., occurring more than 90% of the time), can also feed into a mechanism for identifying when there is a concerning deviation in the number of deaths. Sustained numbers of days with deaths above the forecast can inform a health system about the occurrence of a likely environmental exposure or emerging disease. 

Furthermore, because the forecasts are conditional, relatively low absolute numbers of deaths occurring in the summer, can still trigger a response when those numbers (although low) fall consistently above the conditional 90^th^ percentile. They also forecast when resources and capacity may be reduced.

The analysis presented here showed some forecasting benefit associated with the inclusion of selected lags of daily weather or air quality data (i.e., a difference in R1 between .161 and .194 – a 3% improvement over the unconditional model). A trade-off arises, however, between developing more complex conditional models over models including only temporal and seasonal predictors.

There are important limitations with the approach taken here, and these can be used to highlight future pathways for analysis. The first limitation is with the use of the 90^th^ percentile. One can potentially analyse the data at any conditional quantile, and for different purposes (such as surveillance or resource allocation) analyses at different quantiles – or multiple quantiles – may be more useful. The utility is driven by the application, and as we were seeking a proof of concept, the 90^th^ percentile seemed to be appropriate level. Using cosinor values of yearly and half yearly cycles may not capture important seasonal information that could be built into the forecasts, and is certainly worthy of future investigation. There is a balance to be made in forecasting between the gain in accuracy and the cost of implementation. Sinusoidal functions capturing seasonal and temporal variation are trivial to develop and implement, and provide around a 15-20% improvement in accuracy over using an annual figure for the 90^th^ percentile. More complicated conditional models appear to add a 2-3% improvement. The utility of the gain for the effort is uncertain. The final limitation we consider here is a theoretical one. There is often concern expressed with forecasting models that do not take a more traditional causal modelling approach [31]. We would take two distinct lines of argument in response. The first line of response is that the purpose of forecasting is not about determining cause and effect, and therefore forecasting models should be judged according to their forecasting accuracy, not for their inadequacy at providing causal explanations. The second line of response is that if a causal model out-performs non-causal model in forecast accuracy, then the causal model should absolutely replace the non-causal model. The causal model was not developed here, but there is some reason to believe that it may not perform as well as a “dust bowl” empirical approach [31] that has no interest in explaining the relationships between factors involved but rather for forecasting the outcome.

## Conclusion

This study reports for the first time, a statistical approach for forecasting respiratory related deaths at the 90^th^ percentile using quantile regressions. The results suggest there is potential value in this, even when the model is no more sophisticated than a seasonal/temporal model. The study should, however, be treated as a proof of concept, rather than definitive.
